# Determinants of mental health help-seeking in Europe: a systematic review based on the behavioural model of health services use

**DOI:** 10.1186/s13033-026-00707-y

**Published:** 2026-04-26

**Authors:** Sílvia Conde, Miguel Xavier, Virgínia Conceição

**Affiliations:** 1 National School of Public Health, NOVA University Lisbon, Lisbon, Portugal; 2 Comprehensive Health Research Centre (CHRC), Lisbon, Portugal; 3 National Coordination of Mental Health Policies, Ministry of Health, Lisbon, Portugal; 4 NOVA Medical School, NOVA University Lisbon, Lisbon, Portugal; 5 Institute of Public Health of the University of Porto, University of Porto, Porto, Portugal

**Keywords:** Mental health, Help-seeking, European countries

## Abstract

**Background:**

Help-seeking behaviours for mental health are critical for timely intervention, yet vary widely across populations due to multiple influencing factors. These include demographics, psychological attitudes, sociocultural norms, stigma, and healthcare systems. This review aims to identify factor associated with help-seeking behaviours related to mental health across European countries, guided by Andersen’s Behavioural Model of Health Services Use.

**Methods:**

This systematic review followed PRISMA guidelines, examining quantitative research published between January 2000 and October 2024 across PubMed, PsycINFO, and Web of Science. Studies were eligible if they included only adult participants residing in European countries who reported mental health symptoms or diagnoses. Data extraction and synthesis focused on identifying key barriers, facilitators, and factors associated with help-seeking behaviour.

**Results:**

Seventeen studies, primarily cross-sectional, met the inclusion criteria. Despite a high prevalence of mental disorders, these studies revealed a low perceived need for care. It was found that personal stigma significantly reduces help-seeking, while positive attitudes towards mental healthcare, higher psychological distress, longer symptom duration, and stronger internal locus of control increased help-seeking likelihood. Men, individuals with lower levels of education, and those from conservative social contexts were less likely to seek support. Structural barriers included inadequate social support and geographic disparities.

**Conclusions:**

This review highlights modifiable factors, particularly stigma, mental health literacy and service accessibility. Addressing these through targeted interventions, such as educational campaigns, community-based programs, and early detection strategies, may improve mental health service utilisation across Europe. Future research should focus on longitudinal, intersectional, and culturally-sensitive studies to further refine effective mental health policies and interventions.

## Background

Mental health problems are highly prevalent worldwide, with one in four individuals experiencing mental disorders such as anxiety, depression, and bipolar disorder throughout their lives [1]. According to estimates, the COVID-19 pandemic has contributed to an increase in these figures, leading to a considerable rise in mental health burden. In Europe, post-pandemic estimates show an increase in the prevalence of mental disorders of approximately 5% in countries such as Finland and Ireland, and about 20% in countries such as Croatia and Portugal [2, 3].

Mental health disorders not only affect individuals but also have societal and health system implications. In 2021, mental disorders were responsible for approximately 155 million disability-adjusted life years (DALYs) globally, with an increasing trend observed over the past three decades [4, 5]. Although they are not commonly recorded as direct causes of death in vital statistics, mental disorders, particularly severe conditions such as depression, psychosis, and substance use disorders, are strongly associated with increased premature mortality, often through comorbidities or suicide [6]. According to the Global Burden of Disease 2021 study, there were an estimated 746,000 deaths due to suicide worldwide in 2021, with higher mortality rates in Eastern Europe and parts of Western Europe, including countries such as Portugal, Belgium, and Sweden [6]. These findings underscore the need to address mental health as both a personal and a public health priority.

These statistics challenge European mental health services to address community needs with more responsive, equitable, and accessible services [7]. To improve mental health service delivery in Europe, it is also crucial to understand the patterns and determinants of mental health service help-seeking and utilisation among European populations [8]. This point is especially important since a significant proportion of adults with serious psychological distress or mental health problems do not seek assistance nor receive appropriate treatment [9–11].

Help-seeking behaviour for mental health symptoms is a complex process encompassing symptom perception, interpretation, appraisal, and decision-making [12]. A wide range of individual, social, and structural factors influencing the help-seeking process was identified in the literature. These include demographic variables (e.g. age, gender, education), psychological determinants (e.g. stigma, attitudes, perceived need), and service-related elements such as affordability and accessibility [7]. Determining the factors that contribute more significantly towards help-seeking behaviours is challenging. Much of the research in this field focuses on only a few factors, with most studies rarely being guided by theory-driven hypotheses. By using a theoretical framework, it is possible to summarise different influences in a more parsimonious way. The Andersen Behavioural Model of Health Services Use offers an integrative perspective to capture the broader range of individual, contextual, and structural factors involved in service utilisation [13]. Its structure allows for the classification of determinants across three key domains: predisposing factors (e.g. demographic attributes, beliefs), enabling factors (e.g. access, resources), and need-related factors (e.g. perceived or clinical severity). These features make the model especially suitable for analysing intended and actual service utilisation across diverse European health systems [7]. Furthermore, given the growing policy focus on equity and responsiveness [14], a structured synthesis guided by Andersen’s model can contribute to bridging the gap between empirical evidence and actionable reform.

In this systematic review, we apply Andersen’s model to synthesise the determinants of mental health help-seeking among adults in European countries. By structuring the evidence within a coherent theoretical framework, we aim to enhance understanding of how individual, social, and structural factors shape service use and to inform strategies for improving access and equity.

## Methods

### Study protocol

We conducted this systematic review following the PRISMA (Preferred Reporting Items for Systematic Reviews and Meta-Analyses) guidelines to ensure a rigorous and transparent approach to study selection and synthesis [15]. The protocol was registered in the International Prospective Register of Systematic Reviews (PROSPERO) under registration number CRD42024516820, ensuring methodological transparency and replicability.

### Search strategy

We systematically searched PubMed, PsycINFO, and Web of Science databases to identify quantitative studies published between January 2000 and October 2024. Appendix 1 provides the search syntax, including keywords and Boolean operators, allowing for replication.

### Eligibility criteria

Studies were eligible for inclusion if they met predefined criteria organised within the PI(E)COS framework [16]. Eligible populations included adult individuals (≥ 18 years) residing in European countries (see Appendix 2 for a list of the considered countries) who reported experiencing mental health symptoms or diagnosed mental health conditions. All genders, socio-economic backgrounds, and ethnicities were included. As exposures of interest, we considered mental health service utilisation determinants classified according to Andersen’s Behavioural Model (predisposing, enabling, and need factors). Studies were eligible regardless of whether they included comparator groups.

As primary outcomes, we focused on studies reporting actual or intended mental health service utilisation, with secondary outcomes examining factors such as stigma, knowledge of mental health conditions, and perceived barriers.

Eligible study designs were randomised controlled trials, cohort studies, case-control studies, and cross-sectional analyses published in English, Portuguese, Spanish, or Italian between January 2000 and October 2024.

Finally, we excluded studies that focused exclusively on participants under the age of 18, that were conducted outside Europe, or lacked primary outcomes directly related to help-seeking or mental health service utilisation.

### Data extraction and study selection

In the selection process, we followed a structured approach, outlining the steps from initial identification to screening, eligibility assessment, and final inclusion of the studies. Study metadata was extracted using EndNote. We have removed duplicates using Covidence and screened the remaining titles and abstracts. Two reviewers independently screened the records based on predefined eligibility criteria and resolved any discrepancies through discussion and consensus. Two reviewers independently extracted data using a structured template, provided in Table 3 (Appendix 3). The extracted information included, among other details: author(s), year, country, study design, sample characteristics, outcome measures, help-seeking determinants assessed, and instruments used. Disagreements were settled by consensus. A third reviewer was available for arbitration in cases where consensus was not achieved, but was ultimately not necessary. 

### Risk of bias

The quality of the included studies was assessed using the Quality Assessment Tool for Observational Cohort and Cross-Sectional Studies [17], which is specifically designed to evaluate the risk of bias in observational studies. This tool is a methodological instrument developed by the National Heart, Lung, and Blood Institute (NHLBI) to evaluate the risk of bias and quality of observational studies. It includes a set of criteria that assess key aspects, such as study objectives, study population, sample size justification, exposure and outcome measurements, confounding variables, and the appropriateness of statistical analyses. By systematically addressing these domains, the tool provides a structured approach to determining the internal validity and reliability of observational cohort and cross-sectional studies.

### Data synthesis and analysis

Owing to the heterogeneity in study designs, instruments, populations and reported outcomes, we conducted a narrative synthesis to summarise the findings. We then organised the findings thematically, according to Andersen’s Behavioural Model, grouping factors into predisposing, enabling, and need-related categories.

## Results

### Study selection

**Fig. 1:**
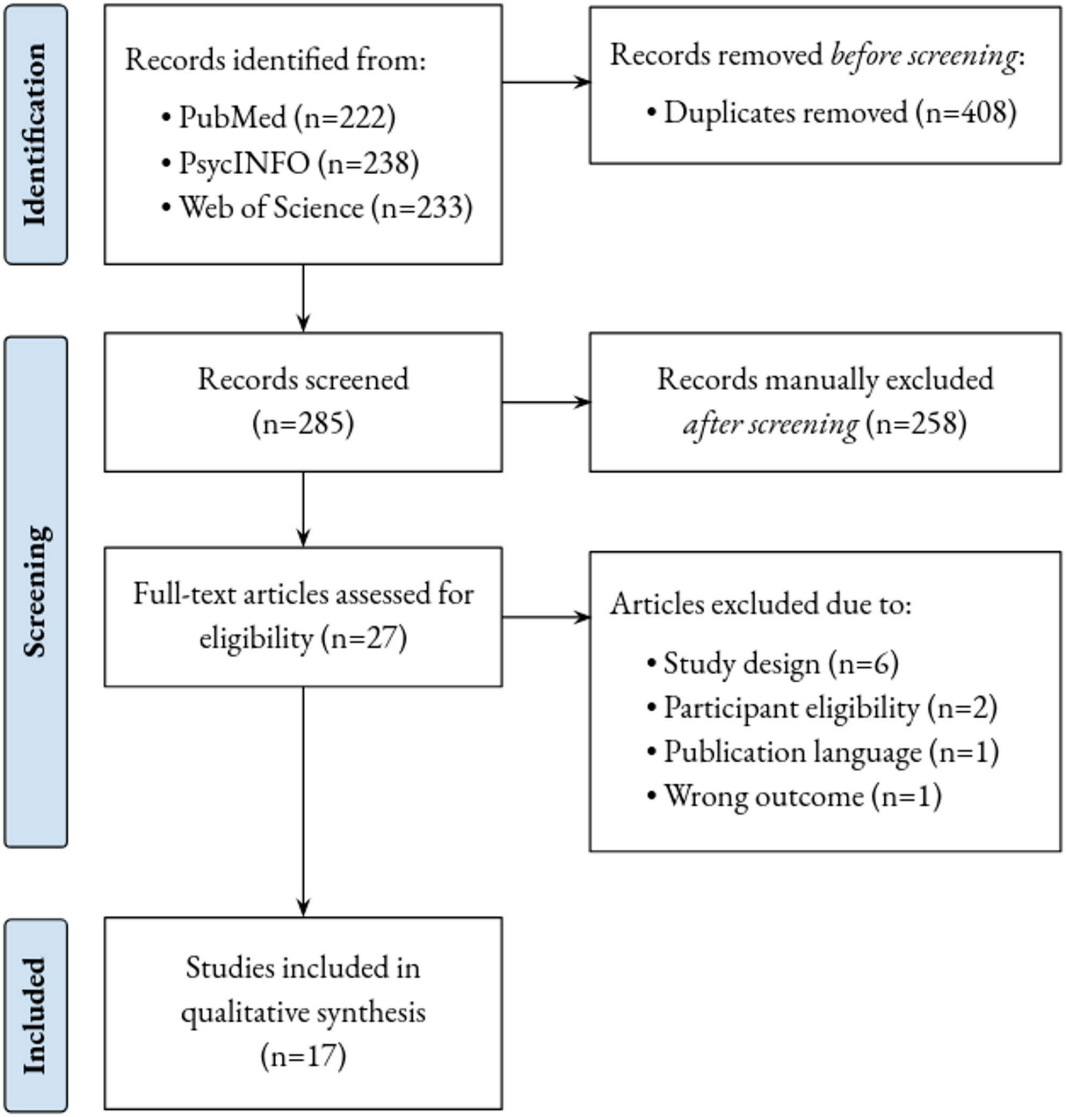
PRISMA flow diagram of the study selection process

The structured approach followed during the selection process is documented in a PRISMA flow diagram (Fig. 1).

We initially retrieved 693 records across the three databases. After removing duplicates using Covidence, we screened 285 titles and abstracts. Two reviewers independently screened all records based on predefined eligibility criteria and resolved any discrepancies through discussion and consensus.

We then assessed the remaining 27 articles by reviewing their full texts. Of these, 17 met all eligibility criteria, while 10 were excluded: six due to incompatible study designs, two based on participant criteria, one for reporting an irrelevant outcome, and one due to language restrictions (see Fig. 1). Inter-rater agreement was substantial at both stages, with estimated Cohen’s kappa values of approximately 0.75 (title/abstract) and 0.87 (full-text).

The selected 17 studies examined mental health help-seeking behaviours and offered insights into the factors that are associated with seeking professional support. The corresponding data were extracted using the template of Table 3, in Appendix 3.

### Risk of bias

Since all 17 included studies followed an observational design, the selected Quality Assessment Tool was appropriate for evaluating methodological quality across the sample. Four studies met criteria for good methodological quality, while the remaining 13 were rated as moderate. We did not exclude any studies due to a high risk of bias. Despite these limitations, we considered the overall methodological quality sufficient to support a robust narrative synthesis. For a detailed assessment of each study, please refer to Appendix 4 and Table 4 therein.

### Study and participant characteristics

We reviewed 17 studies published between 2008 and 2023, with most of them appearing from 2016 onwards. Sixteen studies used cross-sectional designs, and only one applied a longitudinal approach, limiting the ability to analyse changes in behaviour over time. The study and participant characteristics are summarised in Table 1.

**Table 1:** Overview of included studies: design, country, sample size, and participant characteristics

Reference	Study design	Publication year	Country / Region	Sample size	Age range (in years)	Mean age (in years)	Gender distribution
Boerema et al. [18]	Cross-sectional	2016	Netherlands	102	20–88	52.1	46% men 54% women
Kiss et al. [19]	Cross-sectional	2020	Hungary	272	18–84	28.4	21.3% men 78.3% women 0.4% unknown
Reynders et al. [20]	Cross-sectional	2013	Netherlands, Flanders (Belgium)	2999	18–65	NA	NA
Kohl et al. [21]	Cross-sectional	2023	Germany	658	18–65	36	13% men 87% women
Spahlholz et al. [22]	Cross-sectional	2023	Germany	3042	18–94	49.2	47.2% men 52.4% women 0.4% other
Andersson et al. [23]	Cross-sectional	2014	Western Sweden	3981	19–64	NA	45% men 56% women
Sæther et al. [24]	Cross-sectional	2021	Norway	47,478	18–35	NA	31% men 69% women
Alexi et al. [25]	Cross-sectional	2017	Cyprus	196	18–85	34.5	23% men 77% women
Codony et al. [26]	Cross-sectional	2009	Belgium, France, Germany, Italy, Netherlands, Spain (Western Europe)	8796	NA	47.0	48% men 52% women
Olsson et al. [27]	Longitudinal	2021	Sweden	1240	19–64	NA	100% men
Horackova et al. [28]	Cross-sectional	2019	Western Europe, Scandinavia, Southern Europe and Central and Eastern Europe	28,796	NA	74	47% men 53% women
​Verger et al. [29]	Cross-sectional	2009	France	964	18–24	19.4	37.5% men 62.5% women
Perenc and Radochonski [30]	Cross-sectional	2016	Poland	1706	19–26	22.4	36.6% men 63.5% women
Schomerus et al. [31]	Cross-sectional	2009	Germany	2303	NA	NA	47.5% men 52.5% women
Vanheusden et al. [32]	Cross-sectional	2008	South-West Netherlands	2258	19–32	NA	NA
Olsson et al. [33]	Cross-sectional	2020	Western Sweden	3987	19–64	NA	45% men 55% women
Vanheusden et al. [34]	Cross-sectional	2008	South-West Netherlands	2258	19–32	NA	45.9% men 53.4% women 0.7% unknown

NA = Not available

Geographical representation was uneven. Most studies were conducted in Northern and Western Europe, particularly in the Netherlands, Belgium, Sweden, Germany, and France. Fewer studies focused on Southern and Eastern Europe, with limited data from Portugal, Hungary, and Cyprus.

Sample sizes ranged widely, from 102 [18] to 47,478 participants [24]. While large-scale surveys enabled general population-level estimates, smaller studies provided detailed insights into specific subgroups. Eight studies targeted the general adult population, while six focused on university students or young adults. Two studies examined older adults, while the remaining one explicitly included working-age adults outside the university context. The gender distribution was uneven across studies, with most samples including mostly female participants.

### Narrative synthesis

We have identified a wide range of factors influencing help-seeking behaviours across the included studies. Details of the outcomes measured and instruments used within each study are summarised in Table 2. Taking into account Andersen’s Behavioural Model, we find that predisposing factors were the most frequently reported factors (in 10 studies), followed by need-related factors (9 studies) and enabling factors (3 studies). A narrative synthesis of the results is structured below according to these three domains.

**Table 2:** Outcomes measured and assessment instruments used in included studies

Reference	Outcomes		Outcome measurement tools
	Primary outcomes	Secondary outcomes	
Boerema et al. [18]	Help-seeking, factors determining help-seeking behaviour	Comorbidity with anxiety disorders, symptom duration, and stigma	K10, NEO-FFI, Loneliness Scale, DSS, among others
Kiss et al. [19]	Attitudes toward seeking professional psychological help	Levels of perceived stigma, time perspectives	Zimbardo Time Perspective Inventory (ZTPI), Attitudes Toward Seeking Professional Psychological Help Short Form (ATSPPH-S), Stigma Scale for Receiving Psychological Help (SSRPH)
Reynders et al. [20]	Mental health status, attitudes toward help seeking	Intention to seek professional help, self-stigma, shame, perceived stigma, passive coping	SF-36 Mental Health Summary Scale, Self-Stigma of Seeking Help-Scale (SSOSH), Perceived Devaluation-Discrimination Scale, Attitudes Toward Seeking Professional Psychological Help-Scale, Utrecht Coping Scale
Kohl et al. [21]	Intention to seek PT-A, including discussion of private or occupational burden	Stigma-related barriers, psychological safety climate, treatment experience	WHO-5 (Psychological Well-Being), Stigma Subscale (BACE TSS), PSC-4
Spahlholz et al. [22]	Lifetime help-seeking for mental health	Consultation rates for psychotherapists, GPs	Value orientation scale, Political attitude scale
Andersson et al. [23]	Association between general self-efficacy (GSE) and mental health care-seeking behaviour	GSE’s effect on barriers to care and the choice to seek care among those who reported mental illness	Questionnaire, AUDIT scale (for alcohol consumption), loneliness assessment
Sæther et al. [24]	Use of health services and satisfaction with the health services provided by student welfare organisations	Differences in the use of health services between local and non-local students; Differences in satisfaction with health services between students from large and small student welfare organisations	HSCL-25 (Hopkins Symptom Checklist: to measure symptoms of psychological distress; surveys on the use of health services; satisfaction questionnaires on health services)
Alexi et al. [25]	Willingness to seek help for mental illness	Help-seeking behaviours, openness to mental health services, sources of help	Inventory of Attitudes toward Seeking Mental Health Services (IASMHS), Multidimensional Scale of Perceived Social Support (MSPSS), Practical Barriers in Seeking Mental Health Services (PBMHS)
Codony et al. [26]	Perceived need for mental health care	Use of mental health services, sociodemographic	Composite International Diagnostic Interview 3.0 (CIDI)
Olsson et al. [27]	Mental well-being and its association with prior unmet mental healthcare needs	NA	WHO-10 Well-being Index
Horackova et al. [28]	Prevalence of late-life depression across four European countries	Gap in mental health service use: factors associated with depression	Survey items assessing diagnosis or treatment for depression and socio-demographic, social, and health-related factors
​Verger et al. [29]	12-month prevalence of major psychiatric disorders (MDD, AD, and SUD)	Help-seeking behaviours for psychiatric disorders	WHO-CIDI Short Form for psychiatric diagnoses: Sheehan Disability Scale for Impairment
Perenc and Radochonski [30]	Attitudes toward seeking professional psychological help	NA	ATSPPH-SF
Schomerus et al. [31]	Help-seeking intentions for depression	Influence of personal discriminatory attitudes (social distance) and sociodemographic factors (gender, previous psychiatric treatment) on help-seeking	ADSP (anticipated discrimination when seeing a psychiatrist) scale; SDSP (social distance from someone seeking psychiatric treatment) scale; Depressive symptoms; Sociodemographic data
Vanheusden et al. [32]	Use of mental health services (primary and speciality) within the past 12 months	Sociodemographic disparities in service use	Questions on socio-demographic factors; Questions on mental health service use (primary and speciality)
Olsson et al. [33]	Perceived unmet need for mental healthcare (refraining from seeking care or perceiving care as insufficient)	Factors associated with unmet need (gender, education, country of birth)	Questionnaire-based survey; mental well-being indicators (possibly self-reported symptoms of mental health disorders)
Vanheusden et al. [34]	Identification of barriers to seeking help, categorised into denial of problems, perceptions of problems as self-limiting, and negative perceptions of help-seeking; Analysis of which factors prevent young adults from seeking care	Improved understanding of the barriers young adults face in accessing mental health services; Insights into potential interventions to reduce these barriers and improve engagement	Barriers-to-Care checklist: a tool used to assess different types of barriers young adults face when seeking help for mental health problems; Latent Class Analysis: Used to identify patterns and groupings of participants based on their responses to the checklist

#### Predisposing factors

Predisposing factors correspond to individual characteristics that exist before the illness and are associated with the propensity to use health services. These include demographic characteristics, social structure factors (e.g., education, occupation, social class), health beliefs and attitudes (e.g., perceived need, attitudes towards healthcare) and knowledge about health conditions and services (health literacy) [13].

Eleven studies [18–22, 25, 30–34] reported predisposing factors of help-seeking. Across these, gender emerged as a consistently significant variable. In four studies [20, 22, 31, 33], men were significantly less likely than women to seek professional mental health care, even after adjusting for symptom severity or sociodemographic variables. Lower educational attainment was also associated with reduced help-seeking in two studies [22, 33]; however, one study found no significant association between education and perceived insufficient care after adjusting for mental well-being [33].

Attitudinal factors were assessed in most of these studies. Such factors included personal stigma, beliefs about mental health treatment, openness to professional care, and self-stigma. Findings suggest that lower personal stigma is associated with an increased likelihood of help-seeking [18], while positive attitudes toward mental health care facilitate service utilisation [20]. Conversely, stigma acts as a significant barrier to help-seeking [21, 25], particularly in high-suicide rate regions [20]. One study [31] found that self-stigmatisation, rather than anticipated discrimination, posed a greater barrier to help-seeking. In addition, denial of mental health needs and the belief that issues will resolve on their own were identified as deterrents to seeking care [32]. Barriers related to denying problems and believing that issues would be resolved without intervention were also prominent among younger adults [34]. This latter study identified latent patterns of barriers, including negative perceptions of professional help, which contribute to the delay or avoidance of mental health service use.

Help-seeking was also found to be associated with an internal locus of control, with individuals who believe in their ability to manage their outcomes being more likely to seek professional assistance [30]. One study [19] further explored the role of time perspectives, finding that individuals with a more negative or past-oriented outlook were less likely to seek help. Perceived stigma remained a significant predictor of reduced help-seeking intentions, reinforcing findings from other studies on the role of attitudinal barriers.

#### Enabling factors

Enabling factors are the resources and logistical means that facilitate or impede the use of health services. These include income and economic resources (e.g., financial capacity, insurance coverage), accessibility and availability of health services (e.g., geographic location, transportation), organisational aspects of healthcare (e.g., waiting times, appointment availability), and social support networks (e.g., family and community support systems) [13].

Enabling resources were assessed in only three studies [22, 24, 25]. It was found that individuals living in cosmopolitan intellectual social contexts were more likely to seek help than those in more conservative environments [22]. Practical barriers, such as lack of information about services, and the presence of supportive social networks were also identified as significant factors shaping help-seeking attitudes [25]. One study [24] reported that, among students, those with lower perceived social support, particularly non-local students, were found to be more likely to use mental health services more frequently than their counterparts.

#### Need factors

Need factors primarily refer to individuals’ perceived or professionally assessed health problems that directly motivate healthcare utilisation, including aspects such as symptom severity, diagnostic status, and functional impairment [13].

Nine studies [18, 21, 23, 24, 26–29, 32] addressed need-related factors. Concerning assessed needs, mental health disorders are prevalent among university students; however, only 30.5% of the students in need sought professional help [29]. A similar pattern was observed among young adults, where only 34.6% of individuals with mental health problems reported using mental health services [32]. Additionally, older adults, particularly those in Southern Europe, were identified as a high-risk group for late-life depression, while also showing a significant underutilisation of services [28].

In one study [23], 25% of men and 43% of women who reported a mental illness felt they could have benefited from treatment, and, of those, 37% of the men and 27% of the women reported experiencing barriers to care. The same study found that, while low self-efficacy was associated with increased mental health problems, it did not necessarily relate to greater help-seeking behaviour [23].

Despite the prevalence of mental health issues, a significant gap remains in the perceived need for care, which has also been identified as a significant predictor of help-seeking behaviour. One study [26] found that only 33% of individuals diagnosed with a mental health disorder recognised their condition as requiring professional intervention. On the other hand, a poorer well-being was found to be associated with a higher help-seeking intention [21]. Similarly, a longer symptom duration was linked to a greater likelihood of seeking help [18]. Students experiencing severe mental distress were found to use nearly all types of health services more frequently than their peers [24].

Finally, one study [27] highlighted the link between prior unmet mental healthcare needs and reduced well-being, suggesting that a failure to access care when initially needed may contribute to worsening mental health outcomes. This underscores the long-term consequences of barriers to care in at-risk populations.

## Discussion

This systematic review synthesised recent evidence on factors associated with help-seeking for mental health reasons among adults in European countries, guided by Andersen’s Behavioural Model of Health Services Use. The findings confirm that a complex interplay of predisposing, enabling, and need-related factors influences help-seeking behaviour.

### Key themes

Personal stigma emerged as a consistent and powerful deterrent, while openness to care, symptom severity, internal locus of control, and prior experience with services increased the likelihood of help-seeking.

A particularly relevant finding across multiple studies was the discrepancy between clinical indicators of need and individuals’ perceptions of that need. Many respondents, including those reporting mental health symptoms, did not perceive a need for professional help [26]. This misalignment may be partly explained by poor mental health literacy [35], different facets of stigma [31], and beliefs about self-management [32].

Gender differences in help-seeking were another robust finding. Even after controlling for symptom severity, men were consistently less likely to seek help than women, a pattern observed across both older and younger populations [33]. This finding is consistent with the broader literature, which highlights masculine norms that equate help-seeking with weakness or loss of control [36]. Cultural context also played a role: individuals in cosmopolitan, secular environments were more likely to seek help than those embedded in traditional or conservative communities [22].

The recognition of stigma (including self-stigma) as a barrier, and social support as a facilitator, aligns with findings from previous studies, including the systematic review [37], conducted over a decade ago in other parts of the world.

### Implications for policy

While key barriers to help-seeking may be deeply entrenched and resistant to change, this review identifies several modifiable factors that can serve as effective intervention points to improve mental health service utilisation. These include stigma reduction, mental health literacy, and increasing care accessibility. Predisposing factors such as demographics may be less amenable to change; however, attitudes toward mental health, psychological characteristics, and perceived barriers can be influenced through targeted, evidence-based strategies.

#### Addressing predisposing factors

From a policy and practice perspective, our findings indicate the need to address attitudinal barriers. Stigma remains one of the most significant modifiable barriers to help-seeking, in line with prior international findings [38]. This review reinforces the importance of stigma as a predisposing factor that inhibits help-seeking. While positive attitudes toward mental health care were shown to facilitate help-seeking, stigma, denial of mental health conditions, and beliefs that problems would resolve independently acted as strong deterrents. Reducing stigma through culturally adapted public health campaigns and education initiatives is essential.

Targeted anti-stigma initiatives to promote mental health literacy can help normalise the use of mental health services. They could play a pivotal role in increasing utilisation, especially among men, who were found to be consistently less likely to seek help. Peer-delivered outreach as well as mental health promotion efforts specifically designed for men have shown positive results in reducing stigma and increasing service use [39, 40].

Finally, an internal locus of control was also positively associated with help-seeking, suggesting that empowerment-based interventions could enhance individuals’ motivation and readiness to seek care.

#### Addressing enabling factors

Although structural barriers such as geographic access are more difficult to modify in the short term, there is evidence that social support and perceived accessibility play central roles in shaping help-seeking behaviour [13, 41–43]. Interestingly, our findings suggest that individuals with lower perceived social support were more likely to engage with services, potentially due to a greater reliance on formal care when informal resources are lacking. This highlights the importance of institutions providing access to community-based mental health services, particularly for individuals who are geographically or socially isolated. At the same time, it is important to acknowledge that informal support networks (e.g., family, friends, and community ties) can act as protective factors, potentially mitigating distress and reducing the need for formal service use in some contexts.

The influence of cultural context, evident in higher help-seeking rates in secular and cosmopolitan environments, underscores the importance of tailoring interventions to local norms and values. Community-based outreach, peer-led mental health advocacy, and culturally adapted education programs may help reduce resistance to professional care in more conservative populations.

#### Addressing need factors

One of the most concerning findings is the persistent gap between mental health symptoms and the perceived need for care [26, 44]. Despite the high prevalence of psychological distress and diagnosable conditions, only around one-third of individuals with mental health disorders acknowledged needing treatment. This indicates a need for an improved public understanding of mental health, and for proactive screening initiatives, particularly among high-risk populations, such as university students and older adults.

Symptom duration was another important determinant. Several studies found that the longer symptoms persisted, the more likely individuals were to engage in help-seeking behaviours. This points to the value of early detection strategies, such as regular mental health screenings in schools, universities, and primary care settings, to identify individuals in need of support and to reduce chronicity and burden [45, 46].

### Limitations

This review offers several methodological strengths, including a registered protocol, a comprehensive multilingual search, and the application of a theoretically grounded framework. By structuring the evidence through Andersen’s model, we were able to highlight not only widely known factors associated with help-seeking but also neglected factors, particularly enabling factors and broader contextual influences. The inclusion of studies from different European regions also contributes to the generalisability of findings.

Nonetheless, some limitations of the included studies must be acknowledged. The restriction to studies published in English, Portuguese, Spanish, and Italian, together with the use of English search terms, may have led to the omission of relevant studies in other languages (e.g., French), representing a potential source of language bias. The predominance of cross-sectional designs limits the ability to assess causality between determinants and help-seeking behaviour. Additionally, most studies relied on self-reported data, which may be affected by recall bias and social desirability, particularly concerning stigma or willingness to seek care. Integrating objective data sources, such as clinical records or insurance claims, would strengthen the reliability of findings and provide a more accurate picture of actual service utilisation.

Sampling bias was another limitation, as many studies were conducted among university students or highly educated populations, which may not reflect the broader population. This overrepresentation potentially obscures the experiences of older adults, individuals with lower socioeconomic status, rural communities, and gender-diverse populations. Furthermore, cultural and contextual factors were underexamined, with few studies accounting for sociopolitical environments or belief systems. The underrepresentation of Southern and Eastern European countries may reflect broader disparities in research funding and publication visibility, and addressing this gap would be essential for achieving equitable mental health systems across Europe.

We note that economic barriers were insufficiently explored. While some studies acknowledged financial concerns, few investigated the role of healthcare costs, insurance coverage, or mental health policy frameworks. Understanding these structural factors may be relevant for policy and practice targeting low-income and marginalised populations. There is also a notable lack of research on non-traditional interventions, including digital mental health tools (e.g., teletherapy, apps) or peer support programs, an increasingly relevant area given shifts in care delivery.

Finally, the exclusion of grey literature in this review may have omitted valuable insights from community-based or grassroots mental health initiatives that have not yet been published in peer-reviewed journals.

### Future research directions

Our review contributes to the literature by incorporating recent studies from underrepresented European countries and applying a structured, theory-driven framework. However, important geographical disparities remain: most studies were conducted in Northern and Western Europe. This imbalance highlights the need for greater research investment in underrepresented regions to ensure more inclusive and representative evidence.

To build on this work, future research should prioritise longitudinal studies that can explore the temporal dynamics of help-seeking and establish causality. Mixed-methods designs, combining large-scale survey data with in-depth qualitative interviews, would provide richer insights into individual trajectories and professional perspectives. Methodologically, complementing self-reports with objective service utilisation data, using validated instruments, and ensuring high measurement reliability would strengthen the robustness of findings.

Importantly, future studies should examine help-seeking behaviours and intentions related to emerging intervention models, including digital and community-based services, and evaluate their accessibility, acceptability, and impact across different groups. Moreover, future research should seek to include diverse populations, particularly those underrepresented in current studies, and consider intersectional analyses that examine how gender, age, ethnicity, socioeconomic status, and other factors intersect in shaping help-seeking behaviour. Ultimately, expanding research in these areas will support the development of more effective, equitable, and culturally sensitive mental health systems across Europe.

## Conclusions

Despite significant investment in mental health services and increasing public awareness, mental health help-seeking in Europe remains hampered by enduring stigma, low perceived need, and sociocultural norms. Structural improvements in care availability have not yet led to equivalent behavioural change or a reduction in unmet needs.

Addressing both psychological and contextual barriers through equity-focused, theoretically informed strategies is critical. These may include educational campaigns, community-based programs, and early detection strategies. Reliance on a widely accepted theoretical framework such as Andersen’s Behavioural Model of Health Services Use can help bridge the gap between research and policy, translating findings into action and enabling the design of more responsive, person-centred mental health systems.

## Data Availability

No datasets were generated or analysed during the current study.

## Appendix 1. Search keywords and syntax

(“mental health” OR “mental disorders” OR “psychological symptoms” OR “mental illness” OR “depression” OR “anxiety” OR “schizophrenia” OR “bipolar disorder” OR “PTSD” OR “obsessive-compulsive disorder” OR “OCD” OR “eating disorders” OR “personality disorders” OR “psychosis”)

AND

(“help-seeking behaviour” OR “help-seeking” OR “seeking care” OR “access to care”)

AND

(“Austria” OR “Belgium” OR “Bulgaria” OR “Croatia” OR “Cyprus” OR “Czech Republic” OR “Denmark” OR “Estonia” OR “Finland” OR “France” OR “Germany” OR “Greece” OR “Hungary” OR “Ireland” OR “Italy” OR “Latvia” OR “Lithuania” OR “Luxembourg” OR “Malta” OR “Netherlands” OR “Poland” OR “Portugal” OR “Romania” OR “Slovakia” OR “Slovenia” OR “Spain” OR “Sweden” OR “United Kingdom” OR “Norway” OR “Switzerland” OR “Iceland” OR “Liechtenstein”)

AND

(“determinants” OR “factors” OR “influences” OR “barriers” OR “facilitators”)

AND

(“diagnosis” OR “pre-diagnosis” OR “post-diagnosis”)

## Appendix 2. List of considered countries

By European countries, we mean countries belonging to the European Union, or the Schengen Area and the United Kingdom, namely: Austria, Belgium, Bulgaria, Croatia, Cyprus, Czech Republic, Denmark, Estonia, Finland, France, Germany, Greece, Hungary, Iceland, Ireland, Italy, Latvia, Liechtenstein, Lithuania, Luxembourg, Malta, Netherlands, Norway, Poland, Portugal, Romania, Slovakia, Slovenia, Spain, Sweden, Switzerland, and United Kingdom.

## Appendix 3. Data extraction template

**Table 3:** Template for the systematic extraction of study data

General information	Author(s)
	Title
	Journal
	Publication Year
	Year of data collection
	Country (and region, if applicable)
	Study design
	Study period
Participant characteristics	Recruited participants
	Total sample size
	Inclusion criteria
	Exclusion criteria
	Age range
	Mean age
	Gender breakdown
Determinants assessed	Health determinants
	Barrier determinants
Help-seeking patterns and services	Mode of delivery (in-person, online, etc.)
	Mental health service provider (psychologists, peers, etc.)
	Control group intervention (if applicable)
Outcome measures	Primary outcome
	Secondary outcomes
	Measurement tools
	Measurement time points
Results	Key findings
	Main outcomes
	Secondary outcomes
Additional notes	Limitations

## Appendix 4. Quality assessment tool for observational cohort and cross-sectional studies

**Table 4:** Risk of bias analysis

Reference	Study Design	Quality Assessment Criteria														Quality Assessment Rating
		1.	2.	3.	4.	5.	6.	7.	8.	9.	10.	11.	12.	13.	14.	
Boerema et al. [18]	Cross-sectional	Yes	Yes	No	Yes	No	NA	NA	Yes	Yes	NA	Yes	NA	NA	Yes	Fair
Kiss et al. [19]	Cross-sectional	Yes	Yes	No	Yes	No	NA	NA	Yes	Yes	NA	Yes	NA	NA	Yes	Fair
Reynders et al. [20]	Cross-sectional	Yes	Yes	No	Yes	No	NA	NA	Yes	Yes	NA	Yes	NA	NA	Yes	Fair
Kohl et al. [21]	Cross-sectional	Yes	Yes	No	Yes	No	NA	NA	Yes	Yes	NA	Yes	NA	NA	Yes	Fair
Spahlholz et al. [22]	Cross-sectional	Yes	Yes	Yes	Yes	No	NA	NA	Yes	Yes	NA	Yes	NA	NA	Yes	Good
Andersson et al. [23]	Cross-sectional	Yes	Yes	Yes	Yes	No	NA	NA	Yes	Yes	NA	Yes	NA	NA	Yes	Good
Sæther et al. [24]	Cross-sectional	Yes	Yes	No	Yes	No	NA	NA	Yes	Yes	NA	Yes	NA	NA	Yes	Fair
Alexi et al. [25]	Cross-sectional	Yes	Yes	CD	Yes	No	NA	NA	Yes	Yes	NA	Yes	NA	NA	Yes	Fair
Codony et al. [26]	Cross-sectional	Yes	Yes	Yes	Yes	No	NA	NA	Yes	Yes	NA	Yes	NA	NA	Yes	Good
Olsson et al. [27]	Longitudinal	Yes	Yes	No	Yes	No	Yes	Yes	Yes	Yes	Yes	Yes	NA	No	Yes	Good
Horackova et al. [28]	Cross-sectional	Yes	Yes	CD	Yes	No	NA	NA	Yes	Yes	NA	Yes	NA	NA	Yes	Fair
​Verger et al. [29]	Cross-sectional	Yes	Yes	Yes	Yes	Yes	NA	NA	Yes	No	NA	Yes	NA	Yes	Yes	Good
Perenc and Radochonski [30]	Cross-sectional	Yes	Yes	Yes	Yes	No	NA	NA	Yes	Yes	NA	Yes	NA	NA	Yes	Fair
Schomerus et al. [31]	Cross-sectional	Yes	Yes	Yes	Yes	No	NA	NA	Yes	Yes	NA	Yes	NA	NA	Yes	Fair
Vanheusden et al. [32]	Cross-sectional	Yes	Yes	Yes	Yes	No	NA	NA	Yes	Yes	NA	Yes	NA	NA	Yes	Fair
Olsson et al. [33]	Cross-sectional	Yes	Yes	Yes	Yes	No	NA	NA	Yes	Yes	NA	Yes	NA	NA	Yes	Fair
Vanheusden et al. [34]	Cross-sectional	Yes	Yes	Yes	Yes	No	NA	NA	Yes	Yes	NA	Yes	NA	NA	Yes	Fair

CD = cannot determine; NA = not applicable

Quality Assessment Criteria

1. Was the research question or objective in this paper clearly stated?

2. Was the study population clearly specified and defined?

3. Was the participation rate of eligible persons at least 50%?

4. Were all the subjects selected or recruited from the same or similar populations (including the same time period)? Were inclusion and exclusion criteria for being in the study prespecified and applied uniformly to all participants?

5. Was a sample size justification, power description, or variance and effect estimates provided?

6. For the analyses in this paper, were the exposure(s) of interest measured prior to the outcome(s) being measured?

7. Was the timeframe sufficient so that one could reasonably expect to see an association between exposure and outcome if it existed?

8. For exposures that can vary in amount or level, did the study examine different levels of the exposure as related to the outcome (e.g., categories of exposure, or exposure measured as a continuous variable)?

9. Were the exposure measures (independent variables) clearly defined, valid, reliable, and implemented consistently across all study participants?

10. Was the exposure(s) assessed more than once over time?

11. Were the outcome measures (dependent variables) clearly defined, valid, reliable, and implemented consistently across all study participants?

12. Were the outcome assessors blinded to the exposure status of participants?

13. Was loss to follow-up after baseline 20% or less?

14. Were key potential confounding variables measured and adjusted statistically for their impact on the relationship between exposure(s) and outcome(s)?
